# *Nannochloropsis* Lipids and Polyunsaturated Fatty Acids: Potential Applications and Strain Improvement

**DOI:** 10.3390/md23030128

**Published:** 2025-03-15

**Authors:** Sofia Navalho, Narcis Ferrer-Ledo, Maria J. Barbosa, João Varela

**Affiliations:** 1 GreenCoLab—Associação Oceano Verde, University of Algarve, Campus de Gambelas, 8005-139 Faro, Portugal; sofianavalho@greencolab.com; 2 Bioprocess Engineering, AlgaePARC, Wageningen University and Research, P.O. Box 16, 6700 AA Wageningen, The Netherlands; narcis.ferrerledo@gmail.com (N.F.-L.); maria.barbosa@wur.nl (M.J.B.); 3 CCMAR—Centre of Marine Sciences, University of Algarve, Campus de Gambelas, 8005-139 Faro, Portugal

**Keywords:** *Nannochloropsis*, strain improvement, PUFA quantification, PUFA applications, algal lipid analysis

## Abstract

The genus *Nannochloropsis* comprises a group of oleaginous microalgae that accumulate polyunsaturated fatty acids (PUFAs), especially eicosapentaenoic acid (EPA). These molecules are essential for the correct development and health of humans and animals. Thanks to their attractive lipid profile, *Nannochloropsis* is mainly marketed as a feed ingredient in aquaculture. In microalgae of this genus, contents and cellular location of PUFAs are affected by the growth conditions and gene expression. Strain improvement through non-recombinant approaches can generate more productive strains and efficient bioprocesses for PUFA production. Nevertheless, the lack of specific markers, detection methods, and selective pressure for isolating such mutants remains a bottleneck in classical mutagenesis approaches or lipid quality assessment during cultivation. This review encompasses the importance of PUFAs and lipid classes from *Nannochloropsis* species and their potential applications. Additionally, a revision of the different ways to increase PUFA content in *Nannochloropsis* sp. by using classical mutagenesis and adaptive laboratory evolution is also presented, as well as various methods to label and quantify lipids and PUFAs from *Nannochloropsis* microalgae.

## 1. Introduction

The human population is estimated to grow to 10.4 billion inhabitants by 2100, with a concomitant increase in the demand for food, energy, and water resources [1]. Human activities, namely, growing greenhouse gas emissions and inefficient management of natural (e.g., land) and man-made (e.g., wastewater) resources, have a high impact on the environment. These ecological effects might disturb our daily lives in the form of more frequent natural disasters (e.g., drought, depletion of the ozone layer, acid rain, as well as air, water, and soil contamination), extended disease outbreaks, decreased natural habitats, and consequent loss of biodiversity.

Because of these rising environmental and social problems, there is a need for a rapid change from an unsustainable industry to a sustainable one. Microalgae biotechnology can be part of the solution since microalgae produce unique pigments, proteins, carbohydrates, and lipids of great value to industry while fostering nutrient recycling and effluent treatment [2,3]. Microalgae can be grown photoautotrophically, using simple and abundant nutrients in nature such as sunlight and carbon dioxide; or heterotrophically, using simple or complex carbon feedstocks (e.g., lignocellulosic materials and molasses) [4,5,6]. Furthermore, they have higher areal productivity than land crops, can grow in freshwater, seawater, and even wastewater, and can be cultured on arid land [7].

*Nannochloropsis* is a genus of oleaginous microalgae known for the synthesis and accumulation of up to 5% *w*/*w* of eicosapentaenoic acid (EPA) [8]. Under unfavourable conditions, they overproduce triacylglycerols, which could serve as a feedstock for fuel production (e.g., biodiesel) [9,10]. Moreover, the relatively fast growth and efficient nutrient uptake rates make this genus suitable for the treatment of nitrogen- and phosphorus-rich effluents [11,12,13]. They also accumulate other bioactive molecules (e.g., carotenoids, vitamins) whose functional properties could be useful for the cosmeceutical [14], agricultural [15], food [16], and biomedical sectors [17]. However, their high polyunsaturated fatty acid (PUFA) content has established *Nannochloropsis* spp. as promising oil sources for aquaculture applications [18,19].

The accumulation of PUFAs, and specifically EPA, is highly dependent on the *Nannochloropsis* strain and its growth conditions [20]. The most common strategies for lipid induction (nutrient limitation and adverse physical conditions) tend to increase saturated fatty acids (SFA) and triacylglycerols (TAG) contents at the expense of cell growth and PUFAs occurring in membrane lipids. EPA accumulation in *Nannochloropsis* sp. is optimal under favourable growth conditions, while it decreases under adverse environmental conditions [21,22]. Previous work on *Nannochloropsis gaditana* evaluated the accumulation and distribution of EPA between polar and neutral lipids under different nitrogen and light regimes and showed intrinsic constraints to overproduce EPA [23]. An alternative approach to improve EPA productivities lies in the isolation of mutated strains whose carbon distribution is funnelled towards PUFA synthesis and accumulation. The relentless study of lipid biosynthesis pathways [24], combined with an increasing availability of genetic tools (e.g., promoters, markers, transformation platforms, etc.), has facilitated lipid overexpression in *Nannochloropsis* [25,26]. This has resulted in *Nannochloropsis* phenotypes with increased lipid productivities while maintaining high biomass productivities [27]. The use of such improved strains thriving under suboptimal conditions or showing increased productivity could potentially decrease production costs and increase revenues.

As an alternative to metabolic engineering, classical mutagenesis or adaptive laboratory evolution (ALE) have been extensively used in the past to improve microorganisms of industrial interest. Advantageously, these approaches do not require comprehensive knowledge of the lipid metabolism pathways or expensive equipment. Moreover, the haploid nature of *Nannochloropsis* facilitates the isolation and recognition of phenotypes, and the generated mutants would not fall under the restrictions applied to genetically modified organisms (GMO) in Europe. Nevertheless, the need for a selective pressure and/or screening marker for isolating the phenotype of interest is key when using this approach [28]. Currently, the lack of reliable selective pressure methods and markers for PUFAs remains a major bottleneck in isolating *Nannochloropsis* strains with enhanced PUFA contents through classical mutagenesis.

The present manuscript reviews the current knowledge about *Nannochloropsis* lipids, with a special emphasis on polyunsaturated fatty acids. First, an overview of the distinct fatty acids and lipids classes found in *Nannochloropsis* sp. and how this microalgal genus compares to traditional PUFA sources is given. Second, the most promising applications of PUFAs are summarised. Third, the off- and online methods used to analyse and quantify lipids and PUFAs in microalgae are described, with a particular focus on their application in *Nannochloropsis*. Fourth, different strategies for increasing the PUFA content in *Nannochloropsis* sp. are reviewed, ranging from the manipulation of culture conditions to strain improvement by random mutagenesis and adaptive laboratory evolution. Lastly, the manuscript ends with a section highlighting potential future research areas for *Nannochloropsis* PUFA.

## 2. Lipid Classes and Polyunsaturated Fatty Acids

Lipids comprise fatty acyls, glycerolipids, glycerophospholipids, sphingolipids, sterol lipids, prenol lipids, saccharolipids, and polyketides [29,30], and this review will focus on the first three categories. Glycerolipids and glycerophospholipids contain fatty acyls and they are further subdivided into simple and complex lipids based on their complexity. Simple lipids refer to the ones that can be hydrolysed into two products, at most, and complex lipids refer to those that at least three products are obtained [29]. Additionally, lipids are also frequently subdivided into polar and neutral lipids based on their polarity or amphipathic properties [29,31]. Neutral lipids (NL) in microalgae mainly comprise triacylglycerols (TAG) and, to a lesser extent, diacylglycerols (DAG), monoacylglycerols (MAG), and sterols. TAG, DAG, and MAG contain three, two, or one fatty acyl chains, respectively, esterified to a glycerol backbone. Their function is related to energy storage (TAG), serving as a precursor for the de novo synthesis of lipids (MAG and DAG) and signalling (MAG). Polar lipids (PL) consist of one or two hydrophobic fatty acyl chains esterified in the carbon position *sn*-1 and *sn*-2 of a glycerol molecule. The group esterified in position *sn*-3, whether it is a phosphate, carbohydrate, or a betaine moiety, defines the classification of lipids as glycerophospholipids, glyceroglycolipids, or betaine lipids. The group in that position influences the overall polarity of the entire lipid molecule and its function. Because of their amphipathic nature, polar lipids function as structural components of cell membranes but also provide the ideal environment for the interaction and function of cholesterol, transmembrane proteins, and other molecules [32].

**Figure 1:**
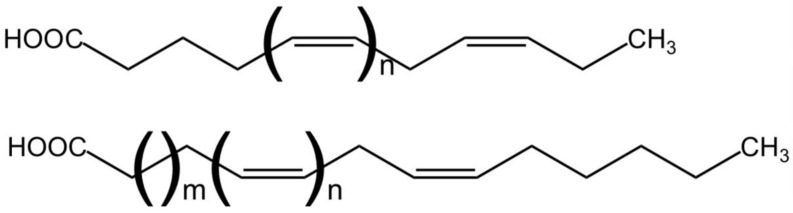
General biochemical composition of ω-3 (top) and ω-6 (bottom) PUFA.

The fatty acids esterified in neutral and polar lipids have variable chain lengths and unsaturation degrees. Based on the unsaturation level, fatty acids are classified into saturated (SFA), monounsaturated (MUFA), or polyunsaturated (PUFA) fatty acids when 0, 1, or more than 1 double bond are present in the acyl chain, respectively. Additionally, unsaturated fatty acids are further subdivided into ω-3, -6, -7, or -9 (also named as *n*-3, -6, -7, or -9) when the first double bond is in the third, sixth, seventh, or ninth carbon counting from the methyl group at the end of the acyl chain, respectively (Figure 1) [33]. While ω-7 and ω-9 fatty acids mainly occur as MUFA, ω-3 and ω-6 are mainly found as PUFAs. PUFAs are usually found in liquid extracts, but *Nannochloropsis* biomass can also be used as a dried PUFA-enriched powder. The solubility of PUFA extracts from *Nannochloropsis* depends on the specific PUFA extracted, the solvent used and the extraction method. Because of their hydrophobic nature, PUFAs are mainly soluble in organic solvents. The extraction of PUFAs from *Nannochloropsis* has already been reviewed elsewhere [34].

The most studied families of PUFAs correspond to ω-3 and ω-6 PUFAs due to their role in the modulation of inflammatory processes and the development of cognitive organs [35]. Supplementation of diets with ω-3 fatty acids has been shown to reduce TAG levels in blood and might lower the risk of coronary heart events, especially in individuals with a history of cardiovascular disorders [36,37,38]. Additionally, their consumption could be associated with a lower risk of specific types of cancer, cognitive function (e.g., Alzheimer), macular degeneration, certain autoimmune diseases (e.g., rheumatoid arthritis), obesity, and osteoarthritis [39,40].

PUFAs are linear hydrocarbon chains where the number of carbon units can range from 14 to 36. For a long time, linoleic acid (LA, C18:2 ω-6) and α-linolenic acid (ALA, C18:3 ω-3) were considered essential fatty acids since they are the main precursors for the synthesis of the remaining ω-3 and ω-6 PUFA. However, the conversion rates of simple carbon precursors to PUFAs are very low in mammals resulting in a need to acquire them externally through diet. LA and ALA are also poorly converted into molecules of higher carbon number and desaturation degree such as arachidonic (ARA, C20:4, ω-6), eicosapentaenoic (EPA, C20:5 ω-3), and docosahexaenoic (DHA; C22:6 ω-3) acids. After LA and ALA, these molecules are of vital importance since they are enzymatically converted to bioactive mediators (eicosa- or docosanoids) with immunomodulatory properties [40]. EPA, DHA, and ARA are occasionally referred to as long-chain PUFAs (LC-PUFAs) since they contain more than 18 carbon atoms. While LA and ALA are present in terrestrial and aquatic biota, LC-PUFAs are commonly found in organisms from marine aquatic habitats such as sea mammals, fish, or algae.

Less knowledge is available regarding the biological function of PUFAs when esterified to a backbone head. PUFAs can be part of either polar or neutral lipids, and the distribution differs between organisms. In photosynthetic organisms, PUFAs predominantly accumulate in the thylakoid membranes, where they are mainly esterified into glycolipids [40]. The presence of PUFAs in membrane bilayers influences the disorder of membranes and therefore their physical properties. PUFAs have been proposed to be the main contributor of membrane fluidity, although recent studies showed that MUFA itself could also fulfil that role [41,42]. PUFAs and MUFAs also influence the thickness of membranes compared to SFA, but PUFAs tend to impart a higher thickness to membranes compared to MUFA due to a longer chain number [42]. Other functions include the alteration of molecule packing as well as the folding and binding of proteins to membrane domains [42]. Polar lipids are responsible for the different membrane organisational domains, namely, lamellar, inverted hexagonal II (HII), cubic, or hexagonal I (HI) phases [43]. Despite the structure of the lipids ultimately influencing the membrane curvature, the elongation and desaturation state of the fatty acids also affect the curvature and organisation of membranes in HII phase [44]. HII phases in phototrophic organisms are responsible for the stacking of grana in the chloroplast of chlorophytes at low light conditions, as well as the correct functioning of enzymatic activities such as the violaxanthin de-epoxidation [43,45]. Finally, PUFAs are natural scavengers of reactive oxygen species (ROS). PUFAs surround the light-harvesting complex of the photosystems while experiencing a highly oxidative environment. In that environment, PUFAs act as natural antioxidants under conditions where ROS are generated such as high light conditions [46].

### 2.1. PUFA Synthesis Pathways

**Figure 2:**
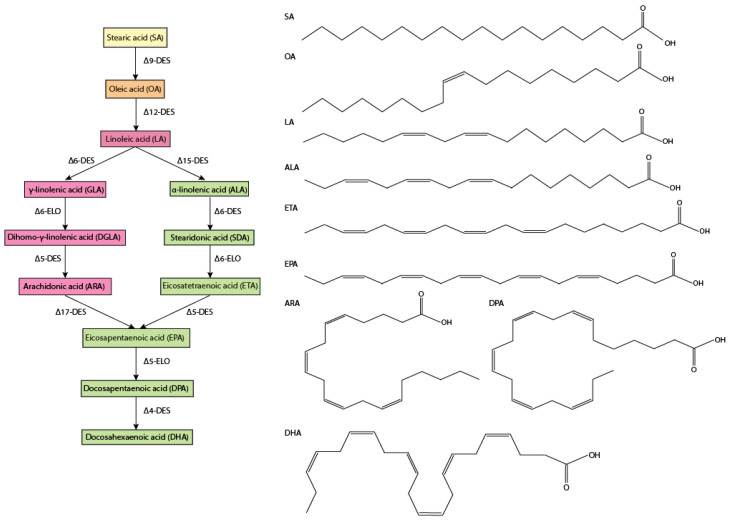
Simplified diagram of the PUFA biosynthetic pathways occurring in the endoplasmic reticulum of microalgae. Enzymes that catalyse reactions in this pathway are mainly desaturases (DES) and elongases (ELO). On the left, saturated (SFA), monosaturated (MUFA), ω-6, and ω-3 polyunsaturated (PUFA) fatty acids are represented in yellow, pink, orange, and green, respectively. On the right panel, chemical structures of the most important fatty acids in this pathway are given.

The biochemical pathways for PUFAs and lipid production in *Nannochloropsis* sp. are known and have been reviewed in detail elsewhere [22,47]. The biosynthesis of long-chain PUFAs in microalgae occurs mainly outside of the chloroplast thanks to the action of different membrane-bound desaturases and one elongase [48,49,50]. As shown in Figure 2, the synthesis of EPA starts from C18:0 (stearic acid, SA), being desaturated to C18:1 (oleic acid, OA) and subsequently to C18:2 (linoleic acid, LA) by the Δ-9 and Δ-12 desaturases, respectively. From this point onwards, LA is desaturated to ω-6 C18:3 (γ-linolenic acid, GLA), following the ω-6 pathway. Alternatively, LA is desaturated to ω-3 C18:3 (α-linolenic acid, ALA) and is therefore routed to the ω-3 pathway [51]. In the ω-3 pathway, ALA is further desaturated and elongated to ω-3 C18:4 (stearidonic acid, SA), ω-3 C20:4 (eicosatetraenoic acid, ETA), and C20:5 (EPA). In the ω-6 pathway, GLA is elongated to ω-6 C20:3 (dihomo-γ-linolenic acid, DGLA), then C20:4 (arachidonic acid, ARA), and then EPA. At this point, both ω-3 and ω-6 PUFAs compete for the enzyme Δ6-desaturase; as a result, excess of either ALA or LA can inhibit the metabolism of the other [52]. Radiolabelling studies showed that the ω-6 pathway would be the most active one for the synthesis of EPA over the ω-3 pathway in *Nannochloropsis* [53].

### 2.2. Occurrence of PUFAs in Nature

Desaturases catalyse the conversion of single-carbon bonds to double-carbon bonds, thus increasing the unsaturation level of fatty acids. A different desaturase is responsible for the position of the double bond insertion, which is reflected in the name of the desaturase. For instance, Δ-12 desaturases catalyse the conversion of oleic acid (OA) into LA, whereas Δ-15 desaturases promote the conversion of LA to ALA [54,55,56]. These enzymes have been identified in plants, lower eukaryotes (such as microalgae) and a few animals including nematodes and insects (e.g., cockroaches and crickets) [57]. The rest of the animals cannot synthesise LA and ALA from SFA, but they can convert these essential FA into LC-PUFAs. However, the low conversion yields still creates the need to obtain those LC-PUFAs through their diet. Primary producers in aquatic and terrestrial ecosystems synthesise and accumulate LC-PUFAs, and these molecules are transferred through the food web to all the other living beings. When these animals do not ingest sufficient ω-3 PUFAs, their growth is decreased. As a result, the availability of these PUFAs plays a role in the overall food web [58].

## 3. Intracellular Organisation of Lipids

**Figure 3:**
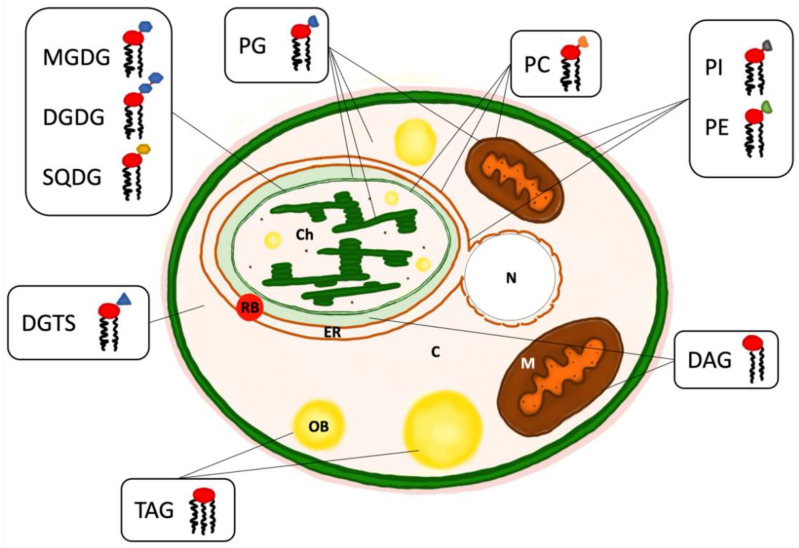
Proposed model of a *Nannochloropsis* cell including the location of the different lipid classes. This model is supported with studies on physiology and cellular organisation of *Nannochloropsis* [73,74,75,76,77]. Organelles—C: cytosol; Ch: chloroplast; ER: endoplasmic reticulum; M: mitochondria; N: nucleus; OB: oil bodies; RB: red body. Lipid classes—DAG: diacylglycerol; DGDG: digalactosyldiacylglycerol; DGTS: diacylglycerol–trimethyl–homoserine; MGDG: monogalactosyldiacyglycerol; PC: phosphatidylcholine; PE: phosphatidylethanolamine; PG: phosphatidylglycerol; PI: phosphatidylinositol; SQDG: sulphoquinovosyldiacylglycerol; TAG: triacylglycerol.

Phospholipids, glycolipids (GL), or betaine lipids (BL) are the main constituents of *Nannochloropsis* membranes (Figure 3). Phospholipids, also known as glycerophospholipids, are composed of a glycerol backbone, a phosphate group, and one or two fatty acids. The specific phospholipid class depends on the functional group attached to the phosphate group. *Nannochloropsis* typically contain phosphatidylethanolamine (PE), phosphatidylglycerol (PG), phosphatidylinositol (PI), and/or phosphatidylcholine (PC) [53]. PC is the most abundant phospholipid in plants and animals, as well as in *Nannochloropsis*. It is ubiquitous in all cellular membranes, except the thylakoids and the chloroplast’s inner membrane. This lipid is highly enriched in C18 fatty acids and serves as a site for the conversion of OA to LA and higher desaturated forms [50]. Alongside playing pivotal in PUFA synthesis and trafficking, it was also shown to be involved in the supply of OA to TAG derived from the chloroplast under nitrogen starvation conditions [59].

After PC, PG is the most abundant phospholipid in *Nannochloropsis.* It is the only phospholipid present in the thylakoid membranes, although in other algae, it was also found in microsomal and mitochondrial membranes [60]. The presence of PG in the thylakoids is indispensable for the correct assembly of photosystems I and II and the correct function of the electron transport chain [61]. Additionally, plastidial PG has the isomeric form C16:1 in *trans*, which was shown to protect the photosystems under oversaturating light conditions in *Nannochloropsis* [46]. PE is the most abundant phospholipid in plants and animals, but this is not the case in *Nannochloropsis* species [8,62]. It is a non-bilayer lipid, and in eukaryotes, it is present in the mitochondria. PE contributes to PUFA biosynthesis by acting as a pool for the desaturation of C20 species to ARA and EPA [49,50].

Glycolipids are made of one or two fatty acids esterified to a glycerol backbone at positions 1 and 2 and a carbohydrate moiety at position 3. They are the most abundant class of lipids in plants, as well as in microalgae, and are located mainly in the chloroplast. The most common types of glycolipids found in photosynthetic organisms are galactolipids, namely, monogalactosyldiacyglycerol (MGDG) and digalactosyldiacylglycerol (DGDG); and sulpholipids, namely, sulphoquinovosyldiacylglycerol (SQDG). The primary difference between these glycolipids is the number and type of sugar molecules they contain, being galactose present in galactolipids and sulphoquinovose in sulpholipids. As well as having a structural and regulatory function in the plastid membranes, glycolipids also play a crucial role in protecting the photosystems from damage and optimizing photosynthesis performance [63]. The size difference of the polar head between MGDG and DGDG affects their structural role, being MGDG a non-bilayer-forming lipid (HII or cubic phase) and DGDG a bilayer-forming lipid (lamellar phase). Moreover, MGDG and DGDG are the endpoint lipid classes for the unsaturated fatty acids EPA and ARA, which influence the membrane organisation and photosynthetic activity [43].

Betaine lipids (BL) are a class of lipids of recent discovery and whose presence is restricted to a few bacteria and protists, including microalgae [64]. Diacylglycerol–trimethyl–homoserine (DGTS), diacylglycerol–trimethyl–beta-alanine (DGTA), and diacylglycerolcarboxylhydroxymethylcholine (DGCC) are the main betaine lipids identified, the last one being the most abundant of the three. Although they are found in the endoplasmic reticulum, they can also be found in other cell locations, such as the chloroplast. Their significance lies in their ability to serve as substitutes for phospholipids under phosphate-scarce conditions [65] or in response to low temperatures [66]. Since this lipid species is highly enriched in EPA, several studies hypothesised a role in supplying EPA to the galactolipids MGDG and DGDG [48,49]. Further studies are, however, required to unveil the role of this lipid class in PUFA biosynthesis.

TAG are the most common simple lipids in microalgae. In the last decades, there has been significant research focused on producing and extracting lipid bodies from microalgae, specifically to use them as a feedstock for biofuel production [67,68]. TAG accumulate under unfavourable conditions to serve as a reservoir of chemical energy inside the cell that can be later used for catabolic reactions. Alongside their role in energy storage, they can also act as a carbon pool for the biosynthesis of membrane polar lipids under optimal conditions and for lipid reshuffling [69]. Under favourable growth conditions, TAG contents in *Nannochloropsis* oscillate around 5% *w*/*w*. Under unfavourable conditions of nitrogen starvation and light irradiance, TAG contents in *Nannochloropsis* can oscillate between 15 and 50% *w*/*w* [70], with a distinct accumulation pattern between species of the same genus. TAG are usually located in intracellular oil bodies (OB), which facilitates their separation and extraction upon cell disruption. OB are cellular organelles that are composed of lipids and other biomolecules, including proteins, and they are associated with lipid biosynthesis and degradation, vesicle trafficking, lipid signaling, and lipid secretion [71,72]. Finally, MAG and DAG are building block molecules used for the synthesis of TAG, but they also serve as precursors for membrane lipids.

## 4. PUFA Applications

The bioactivity of PUFAs—and therefore their final application—significantly depends on the glycerolipid to which they are esterified. For instance, a higher digestibility of PUFAs esterified to polar lipids was reported by in vitro studies using models mimicking the intestinal tract [78,79]. Lipids can also be used as low-molecular-weight emulsifiers or microencapsulates due to their amphipathic nature. The esterification of PUFAs in lipids opens a new space of functionalities to the extracted oils for the food or feed sector, among others. For instance, the emulsification and microencapsulation of PUFAs extends their shelf life, allowing for their application as nutraceuticals or cosmeceuticals. Microalgae such as *Nannochloropsis* can be a sustainable source of PUFA-enriched lipids due to their high contents in EPA, and in decreasing order of quantity, other PUFAs such as ARA, LA, and trace quantities of ALA. The total content of PUFAs in *Nannochloropsis* sp. can go up to 5% *w*/*w* dry weight [80,81]. Feed, cosmetics, nutraceutical, and pharmaceutical applications of microalgal PUFAs have been considered as the most promising for commercial exploitation.

### 4.1. Nutrition: Food and Feed

#### 4.1.1. Food

A diet with a well-balanced ratio of ω-6 and ω-3 (ω-6/ω-3) is very important for a healthy lifestyle. Diets that have a high ω-6 content have been linked with constriction of blood vessels, clumping of platelets, and inflammation [82]. While acute inflammation is a normal response that helps to protect the body against injuries and infections, excessive inflammatory stimuli provide a favourable microenvironment for tumours. Prolonged inflammation has also been associated with a loss of organ functions [83,84]. On the other hand, ω-3 PUFAs have been associated with the induction of apoptosis in tumoral cells, prevention of cardiovascular diseases and anti-atherosclerotic effects [84,85]. A proper intake of ω-3 is also very important for the neural development and correct functioning of brain tissue in infants, as well as for decreasing the risk of cognitive decline and Alzheimer’s disease in adults [86]. Because of this, appropriate amounts of ω-6 and ω-3 fatty acids at a ratio of 4-1 are recommended as an adequate intake and need to be considered in dietary recommendations [87,88].

Because of the limited sources of ω-3, their intake is usually inadequate, contrasting with that of ω-6. Most vegetable oils and crop seeds have low proportions of ω-3 FAs compared to ω-6, as shown in Table 1 [89]. ω-3 are traditionally obtained from the consumption of fatty fish, such as salmon, shellfish, or dietary supplements, such as fish or krill oil [90]. Since there is a finite supply of these marine resources, there is an urgent need to find sustainable PUFA replacement sources for humans’ diets, and microalgae are a well-balanced option [91]. *Nannochloropsis* is a rich source of PUFAs, with EPA lipid contents that are similar to fish oil products (Table 1). Outside Europe, enriched oil extracts from *Nannochloropsis* are already being sold as food supplements, given their rich EPA content (AlmegaPL^®^ or OMEGA-3).

##### Meat Quality

The inclusion of ω-3 FAs in animals’ diets can lead to a higher ω-3 FA content in animals’ meat [93]. Different diets and feeding methods were tested on pigs, and it was found that the meat with the highest nutritional value was from pigs fed with pasture and acorns grazing in an open space [94]. In a study where Mahabadi goats were fed with different fat additives (palm, soybean or fish oil), it was discovered that fish oil supplementation led to a higher DHA and EPA concentration in the muscle meat, with a consequent decrease in the ratio of ω-6/ω-3 [95]. The diets of pigs coated with fish oil for an extra supplementation of PUFAs, with the purpose of preventing the oxidation of the PUFAs, have also been assayed. It was concluded that diet supplementation with PUFA-enriched oils led to a significant decrease in drip loss in the meat of the pigs, as well as better growth of pigs. In addition, a different study concluded that a diet enriched in DHA favours an increased deposition of DHA and total ω-3 fatty acids in different tissues [96]. A few promising studies where microalgal PUFAs were included in diets have been performed. DHA-rich microalgae were used to supplement the diet of lambs, and an improved fatty acid profile of the intramuscular fat was observed, as well as a higher proportion of DHA and ω-3 FA in the muscle tissue. Once again, a diet supplemented with high contents of ω-3 led to enhanced nutritional properties of the meat, with more favourable PUFAs/SFAs and ω-6/ω-3 ratios [97].

##### Dairy

A study involving the use of *Nannochloropsis gaditana* in chicken diets showed the biomass of this microalga can change the fatty acid composition in egg yolk. The microalgal EPA was converted to DHA, which accumulated in the egg phospholipids [98]. Another study tested hem diets with 15% flaxseed, 8% *Schizochytrium* sp. or both (flaxseed + *Schizochytrium*). Flaxseed increased ALA (72%), while *Schizochytrium* increased EPA, DPA and DHA. Both combined achieved the highest ω-3 enrichment [99]. Although *Nannochloropsis* was not tested in this study, the results suggest that it could be a suitable candidate for similar investigations.

Research on cow diets with *Schizochytrium* sp. resulted in changes in the FA composition of the milk, including higher levels of conjugated *cis*-9 *trans*-11 linoleic acid (CLA), *trans*-9 *cis*-11 CLA, oleic acid (OA) [100], and DHA with a decreased fat content [93]. In a different study, cows were supplemented with different protein sources: soybean, *Spirulina platensis*, *Chlorella vulgaris*, and a mixture of *C. vulgaris* and *N. gaditana*. *C. vulgaris* and *N. gaditana* inclusion in the diet resulted in the milk with the most favourable ω-6/ω-3 for human nutrition and a four-fold increase in the content of EPA, without adverse effects on milk’s fat [101]. The existent research in cow’s feed supplementation with *Nannochloropsis* is still in its infancy, but the studies that have been undertaken point to a more complete milk with a balanced FA composition.

#### 4.1.2. Feed in Aquaculture

As well as lipid deposition in food products, the supplementation of PUFAs in aquaculture feed diets has an active role in balancing animal health and welfare [102,103]. Indeed, aquafeed diets must also contain an adequate ratio of ω-6 and ω-3 fatty acids for the survival of fish larvae and juvenile fish and their subsequent growth and development. EPA and DHA are considered the most relevant ω-3 fatty acids in food formulations and must also be supplied in an optimal ratio. The ratio of both ω-3 and ω-6, as well as the one EPA and DHA, is not only species-dependent but also varies with the development stage of the fish [104]. For instance, it was shown that DHA is indispensable at early larvae stages for correct neural and visual development. Fish and krill oils are rich sources of EPA and DHA and are extensively used to supplement the oil fraction of feed diets. Nevertheless, their use poses a societal, environmental and economic threat since they are finite sources essential for maintaining marine trophic webs. Plant-based oils barely contain LC-PUFAs and are not a suitable replacement, while marine microalgae sources are regarded as a good alternative. *Nannochloropsis* is viewed as a natural source of EPA to enrich the ω-3 fatty acid profile in fish diets. Moreover, multiple studies incorporated *Nannochloropsis* sp. in fish meals, some as a substitute for fish flour, others as a supplement to enrich the fish’s diets. Table 2 shows the summarised results of some of these studies.

Lastly, microalgae contain carotenoids, phenolic compounds, or vitamins that can positively impact animal health and well-being by boosting their immune response and increasing disease resistance by protecting against bacteria. They also contain amino acids, among other bioactive compounds, with antioxidant, anti-inflammatory, anti-viral, and photoprotective properties. Additionally, microalgae can also contribute to higher feed conversion and weight gain [91].

**Table 2:** Aquaculture feed and supplementation studies made with *Nannochloropsis* sp.

Animal Species	Microalgae Species	Feed Incorporation (%)	Effect	Reference
Juvenile turbot (*Scophthalmus maximus* L.)	*Nannochloropsis* sp.	2.5, 5, 7.5 and 10	Improved weight gain, enhanced antioxidant capacity, increased digestive enzyme activity at 5% incorporation.	Qiao et al. [105]
Pacific white shrimp	*Nannochloropsis* spp.	0.5, 1, 2	At 1% incorporation, a trend towards lower mortality and increased ROS production was observed. At 2% incorporation, there was a significant increase in resistance to thermal shock and lower mortality and highest production of ROS.	Guimarães et al. [106]
Senegalese sole (*Solea senegalensis*)	*Nannochloropsis gaditana*	3	Increased dry weight; improved growth performance; slight decrease in glutathione levels.	Peixoto et al. [107]
Nile tilapia (*Oreochromis niloticus*)	*Nannochloropsis oculata*	5, 10	At 5%, improved growth performance, increased crude protein, enhanced lipid profile (higher high-density lipoproteins, lower low-density lipoproteins). At 10% further improvement in growth performance, significant increase in ω-3 PUFA, better antioxidant response, higher EPA and DHA content.	Zahran et al. [108]
Kuruma Shrimp (*Marsupenaeus japonicus*)	*Nannochloropsis* sp.	1, 4, 7	A 1% improved survival rate and increased body weight compared to control diet; 4%, alonside increased body weight, also improved feed efficiency and body lipid content; 7% incorporation increased stress tolerance and improved fatty acid profile with higher EPA and DHA.	Adissin et al. [109]

#### 4.1.3. Cosmeceuticals

PUFA deficiency increases proliferative keratins (K6 and K16) and inflammation-related keratins (K17), and they play an important role in epidermal homeostasis [110,111]. More specifically, it was shown that DHA and EPA have protective effects against UV after increasing the minimal erythema dose (MED), which is the minimal dose of UV-B radiation that causes sunburn within 24 h after exposure [112,113,114]. Another study proved the efficacy of *N. oceanica* in combating oxidative stress caused by ultraviolet B radiation. The keratinocyte cells treated with the lipidic extract of *N. oceanica* exhibited increased levels of free and esterified DHA and EPA [115]. An extract from *Nannochloropsis* sp. G1-5 containing fatty acids, carotenoids, and phenolic compounds was investigated in vitro for different activities of interest in cosmetics. The study highlighted the high content of phenolic compounds compared to other microalgae and confirmed a moisturising, anti-wrinkling, and protective effect against UV-B and ROS scavenging activity by inhibiting cell death and tyrosinase activity, respectively [14]. Despite more research being required to understand the skin benefits of microalgae extracts, the use of *Nannochloropsis* extracts is already promoted in therapies such as thalassotherapy for physical and emotional human wellness [116,117,118,119,120,121,122,123].

#### 4.1.4. Pharmaceutical Applications of PUFAs Obtained from *Nannochloropsis* sp.

*Nannochloropsis* extracts containing PUFA-enriched polar lipids have interesting bioactivities that can be of interest for pharmaceutical applications. One study demonstrated in vitro that *Nannochloropsis granulata* has anti-inflammatory properties by evaluating the inhibition of inducible nitric oxide synthase (iNOS) activity, the enzyme responsible for nitric oxide production. This study evaluated six diacylglycerol–trimethyl–homoserine (DGTS) forms in these microalgae, which showed inhibitory activity against nitric oxide production in macrophage cells [124]. This observation was confirmed by another study with *Nannochloropsis oceanica*, which found that DGTS and a phospholipidic extract downregulate iNOS activity [125]. In another study, an extract from *Nannochloropsis* sp. was shown to prevent atherosclerosis by inhibiting oxidative processes and enhancing the protective functions of high-density lipid proteins and related enzymes [126]. Also, in this study, a form of DGTS (lyso-DGTS) was responsible for the inhibitory activity, emphasising the promising applications of this lipid class.

Compared to in vitro studies, in vivo studies using lipids from microalgae are still quite scarce. As a preventive health therapy, a PUFA-enriched oil extracted from *Nannochloropsis oculata* named Almega PL^®^ has already been commercialised due to the bioactivity claims of their polar lipids. A three-month study with Almega PL^®^ supplementation to healthy people showed a 25% decrease in very-low-density lipoprotein cholesterol without increasing low-density lipoprotein cholesterol [127]. These studies show the potential of *Nannochloropsis* sp. extracts as sources of natural bioactive and antioxidant lipids with pharmaceutical applications to prevent and/or treat multiple diseases. Nonetheless, there is still a need to perform more studies in vivo, which is needed for further studies in humans and subsequent product development.

## 5. Detection and Analysis of Lipids and PUFAs

The PUFA content varies with the *Nannochloropsis* species, strain, and growth conditions. The availability of tools for analysing PUFA qualitatively and quantitatively in the whole oil fraction in separate oil fractions or different lipid classes is important. Different methods exist of variable complexity, principle, and measuring time (offline or online), which can specifically target PUFAs or the fractions where PUFAs are located. For instance, online monitoring tools determining the FA profile of the microalgal culture would be useful in defining the optimal harvesting time. Additionally, such online methods hold potential as screening tools to identify strains with enriched PUFA contents. In this section, the most representative methods currently in use are evaluated in terms of their operation and application (Table 3).

### 5.1. Offline

#### 5.1.1. Titration Methods

One of the most widely accepted approaches for assessing the degree of unsaturation in oil samples is through the determination of the iodine value (IV). Despite providing a qualitative assessment, this method has been standardised specifically for measuring the unsaturation degree in vegetable and animal oils (according to ISO, DIN, EN, or AOCS). The unsaturation content provides relevant information on the oxidative quality of the oil before its use in food applications. The method consists of determining the amount of iodine that reacts with the carbon double bonds by titrating a previously weighted oil sample compared to a blank. The titration is commonly undertaken with the Wij method, which involves the use of halogenated solvents such as iodine monochloride (ICl) and time-consuming steps. Regarding the toxicity of the method, attempts were pursued to substitute the toxic solvents used with less harmful ones, such as ethanol [128]. An alternative procedure to titration methods is the definition of the IV number from the fatty acid profile [129] or from spectroscopic methods [130]. In the former approach, the calculated IV is more accurate since the double bonds are clearly defined. Among other applications, the IV is also used to evaluate the suitability of microalgae-derived oils for biodiesel production [131,132].

#### 5.1.2. Colorimetric Methods

An alternative to titration methods is colorimetry. Colorimetric methods are relatively simple, do not require expensive equipment, and can qualitatively evaluate the contents of PUFAs. Nevertheless, the number of colorimetric tests specific to PUFA detection is rather small. One example involves the use of the dye 2,3,5-triphenyltetrazolium chloride (TTC). This compound is colourless in its original state and becomes red after reduction to triphenyl formazan (TF) by specific dehydrogenases [133]. Despite the low specificity of the method, this technique was successfully used to isolate EPA-producing marine bacteria [134] and ARA-producing fungi [135]. A drawback of this method lies in the low stability of TTC to light, which might limit the use of this method in photoautotrophic microorganisms. The sulphophosphovanillin (SPV) method is another example of colorimetric method that has been employed in the screening of oleaginous microorganisms [136,137]. Shortly, the method is based on the pink colour that appears after reacting a carbonium ion (originating from the reaction of a fatty acid and sulphuric acid) and an aromatic phosphate ester (originating from the reaction of the vanillin and the phosphoric acid). The colour intensity depends on the chemical composition of the fatty acid reacting, being more intense with an increasing presence of double bonds. Despite the possibility of measuring PUFA, this method has been mainly tested for the quantification of the lipid content in *N. oceanica* and Thraustochytrids and validated with gas chromatography methods [136,137]. A drawback of this method is that its accuracy is dependent on the reference oil used for calibration.

#### 5.1.3. Fluorometric Methods

After titration and colorimetry, fluorometric methods are the most popular and simplest for quantifying microalgae lipid contents. Fatty acids, including PUFAs, do not emit fluorescence per se, and specific dyes are necessary to stain the lipids of interest. Nile red (NR) and BODIPY (BP) are two popular dyes that have been widely used for the staining of lipid droplets in different oleaginous microalgae. The use of NR and BP can be extended to industrially relevant microalgae genera such as *Nannochloropsis* [138,139], *Tetraselmis* [140], *Scenedesmus* [141], or *Tisochrysis* [142]. Despite the wide use of these dyes for staining neutral lipids, they can also be used for staining membrane lipids. The interaction of the dye with the lipid environment generates an emission spectrum that shifts according to the polarity changes of the environment (solvatochromism). The fluorescence emitted is directly correlated to the level of disorder of membranes, or in other words, the ratio of unsaturated fatty acid species over saturated species in membranes. NR is an example of solvatochromic dye, which was used to infer changes in polar and neutral lipids based on changes in the emission spectra [143,144,145]. The PUFA content and location between the neutral and polar lipid fractions can greatly differ between microalgae species. In one study, the fluorescence emission peaks for polar and neutral lipids, after staining with NR, were used to interrelate changes in the polar and neutral lipids, as well as in the contents of PUFA sin *Tetraselmis suecica* [140]. The polar lipid fraction represents a substantial depot of PUFAs and therefore a relevant target for staining. In a different study with *Nannochloropsis oculata*, the fluorescence signal for polar lipids was overcome by the signal derived from neutral lipids, highlighting difficulties in staining polar lipids in some microalgae [142]. Moreover, the autofluorescence derived from chlorophyll might also spill over a region of interest, thus interfering with the quantification of PUFAs. Therefore, despite the potential of solvatochromic dyes [146], their application for the screening of PUFAs producers is rather limited.

#### 5.1.4. Gas Chromatography

Up to now, analysis by gas chromatography (GC) is still the most reliable and accurate method to identify and quantify the FA profile in a lipid sample. The principle of this method involves the derivatisation of fatty acids into methyl esters and subsequent analysis by gas chromatography (GC) coupled to a flame ionization detector (FID) or mass spectrometer (MS). Despite the importance of this method in the characterisation of the FA profile for many microalgae, there is still information that cannot be retrieved from the GC spectrum (i.e., position of the double bonds). In that case, the combination of GC and MS can be used to obtain further information on the structure of the methyl esters analysed. New variances of MS, such as matrix-assisted laser desorption ionization (MALDI) or electrospray ionisation (ESI), have permitted the analysis of entire lipid molecules without the need for derivatisation. The combined use of MS with high-performance liquid chromatography (HPLC) represents a valuable tool for obtaining information regarding the stereospecific positioning of FA in the glycerol backbone. The use of specific standards can also help in the quantification of lipid classes. Despite the promising results, this technology is still expensive and requires expertise in the topic, being sometimes unaffordable for general use.

#### 5.1.5. Mass Spectrometry

Mass spectrometry represents the most sophisticated technique for lipid analysis, allowing for a comprehensive detail of lipid classes down to a molecular level. When using MS, two different approaches exist: targeted approach, for detection and quantification of known lipid classes in the sample; or untargeted approach, for the identification of new lipid classes. In direct infusion mass spectrometry (DI-MS), lipid samples are directly ionised and analysed without prior separation with limited, and it is typically used for the targeted analysis of lipid classes of interest in a lipid sample [147]. Despite the speed of the analysis, this technique has a limited selectivity and sensitivity towards isomeric lipid class detection. Some of these disadvantages can be overcome by fractionating lipid samples by liquid chromatography (LC). Different types of LC exist, being normal phase (NP), hydrophilic interaction (HILIC), and reverse phase (RP). Liquid chromatography is the most frequently used for lipids. While RP-LC is commonly used for samples containing mainly simple or both simple and complex lipids, NP and HILIC are more appropriate for separating mainly complex lipid samples. Thin-layer chromatography (TLC) can also be used to manually separate and identify the major lipid classes of a microorganism. The inclusion of a chromatographic step allows for a reduction in ion suppression and a broader recognition of the lipidome present in a sample. LC-MS studies have been previously used for evaluating the lipidome of *Nannochloropsis* [148] and how it changes under different nitrogen, phosphorus, and light irradiance conditions [65]. Despite the detailed information obtained from LC-MS, the identification of lipid classes is not straightforward, and sometimes the use of tandem MS (MS/MS) is necessary to characterise the different fragmented products in untargeted approaches [147,149]. MS techniques can also be used for lipid quantification, but the accuracy of the results still remains a challenge. The accuracy of MS for lipid quantification depends on the ionisation efficiency of the lipid classes as well as the availability of specific standards for each lipid class. To solve these discrepancies, methodologies are continuously under development, such as the use of external standards measured by TLC-GC/FID [150]. In Table 4, it is possible to see *Nannochlosopsis* sp. lipid classes composition measured by two different methods LC-MS/MS and TLC-GC/FID.

#### 5.1.6. Vibrational Spectroscopy

Vibrational spectroscopy is a technique primarily employed in the food industry for the nutritional analysis of samples. This technique is widely used in the oil sector to determine the content of fatty acids in *trans* conformation following hydrogenation [151]. The principle of this technique lies in the vibrational changes occurring after absorption of infrared radiation (IR). The induced molecular vibrations provide information on the identity and structure of a molecule, as well as the interactions of a molecule with its surroundings. Moreover, the use of different regions of the IF spectrum (far-infrared, mid-infrared, and near-infrared) can yield different results and, therefore, different applications. When analysing complex samples (i.e., total biomass), IR measurements are supported with the Fourier transform and a broader spectrum is analysed (FTIR). The combined use of IR and multivariate data analysis has allowed the profiling and quantification of the fatty acids in oil samples from microbial sources [152,153]. Despite the advantages of this technique (fast, non-invasive, solvent-free), FA datasets are still necessary to calibrate the chemometric models. In any case, this has not limited the application of IR for the measurement of the FA composition in microalgae [154], or even to monitor the growth of biomass in pilot-scale cultivation systems [155]. This technique was also used in *Nannochloropsis* species. The content of lipids and the most abundant FA in *Nannochloropsis oceanica* (C16:0, C16:1, C20:4; and C20:5) were successfully quantified in pre-treated samples of biomass by using attenuated total reflectance FTIR (ATR-FTIR) [156].

### 5.2. Online

For certain applications, techniques that provide information on the PUFAs profile rapidly, reliably, and with minimal treatment are preferable. Such techniques range from measuring PUFAs in individual cells to the whole culture by indirect or direct approaches. Compared to the offline methods, in situ measurement of PUFAs in microalgae is still in its infancy.

#### 5.2.1. Autofluorescence and Fluorescence from Dyes

Despite the lack of autofluorescence emission by lipid molecules, fluorescence spectroscopy was used to assess the unsaturation degree of the different lipid fractions in microalgae. In that case, statistical methods are needed to decompose the fluorescence signal originating from the interaction of both intracellular fluorophores and non-fluorophores, such as PUFAs. Multivariate data analysis was used to simplify and define the most relevant wavelengths from a fluorescence emission spectrum generated by specific intracellular components such as TAG or membrane lipids. Combined with multivariate regression, models were developed to describe the cell concentration or the pigment contents [157]. By applying projection to latent structure (PL) regression, predictive models were developed for *Nannochloropsis oceanica*, which were capable of predicting the content of EPA and the location of EPA between the polar and neutral lipid fractions at distinct temperatures [158]. In a different experimental dataset, models predicting the cell concentration, the chlorophyll content, and the saturated and unsaturated fatty acids were developed with good predictive accuracy [159]. Such technology holds potential in the real-time monitoring of process parameters related to the lipid quality of the culture [160].

#### 5.2.2. Fluorescence Activated Cell Sorting

Another spectrofluorimetric technique is fluorescence-activated cell sorting (FACS). FACS combines flow cytometry for detecting and analysing the scattering properties of single cells and sorting capacity to separate and isolate the cells of interest. The careful design of the optics and fluidics converts FACS instruments into excellent tools for the high-throughput screening of interesting phenotypes in strain selection programs. When lipids are the target screening molecule, extrinsic fluorophores must be transported inside the cells. As previously discussed, there is a low to inexistent availability of specific fluorescent markers for PUFAs. Nevertheless, this has not limited the use of FACS for quantifying intracellular lipids. Südfeld and coworkers used BODIPY, a dye specific for the staining of lipid droplets, to stain *N. oceanica* cells at different growth stages [33]. FACS has also been used more specifically for screening *Nannochloropsis* cells with increased contents of PUFAs. In that line, Sharma and coworkers combined the use of UV-C and flow cytometry, using NR as a staining agent, to screen and isolate cells with increased contents in EPA [161]. Similarly, Doan and coworkers isolated high lipid producers of *Nannochloropsis*, including EPA, after three rounds of screening and sorting with FACS [162]. Despite the existence of successful examples of the screening and isolation of high PUFA producers, there is still a lack of procedures and methodologies that can identify this phenotype via flow cytometry.

#### 5.2.3. Raman Spectroscopy

Raman spectroscopy is a technique that can be directly used in the measurement of algal samples in vivo. The principle of Raman spectroscopy lies mainly in the measurement of inelastic scattered radiation (also so-called Stokes Raman scattering) that occurs after irradiating a sample with a monochromatic laser beam. The different scattered photons are then represented in a spectrum, where the different peaks provide information on the structure, as well as the chemical composition of the sample. An important advantage of Raman spectroscopy is the low interference of water on the overall signal, highlighting the suitability of this technique for aqueous samples, including microalgal cultures. Nevertheless, the autofluorescence present in the cells interferes with the overall signal, representing a minor setback of this technique. Despite this drawback, numerous studies reported the use of Raman spectroscopy for the intracellular determination of the FA profile in microalgae. Wu and coworkers analysed in vivo the Raman spectra at the cell level of different microalgal species and estimated their degree of unsaturation [163].

An analogue technology to Raman spectroscopy involves the use of terahertz (THz) spectroscopy. In that case, infrared light in the range of 0.1–10 THz is used to excite and stretch hydrogen bonds, generate torsions and vibrate the atoms contained in molecules. It is also a non-invasive technique that has been successfully used in the quantification of different fatty acids [164].

## 6. Strategies to Modulate PUFA Content in Polar and Neutral Lipids in *Nannochloropsis* sp.

There are different ways to increase the PUFA content of microalgae. For example, it is possible to change the culture conditions to trigger the production of fatty acids or apply strain improvement techniques to generate strains with higher contents.

### 6.1. Culture Conditions

Most studies on *Nannochloropsis* lipids focused on the accumulation of fatty acids, and poor attention was given to their distribution between different fractions or even lipid classes [165,166,167,168,169]. In the last decades, the understanding gained on lipidomics techniques has permitted their use for studying microalgal lipids and, more specifically, *Nannochloropsis* lipids. Up to today, few studies on the lipidome of *Nannochloropsis* are available, and it was mainly studied at different light, temperature and nutrient conditions. Even fewer studies are present for other relevant process parameters such as salinity or pH.

#### 6.1.1. Temperature

The lipid content and composition of *Nannochloropsis*, as well as its growth, are highly influenced by temperature. Temperatures lower or higher than the mesophilic conditions reduce cell growth and limit the productivity of the culture. If temperatures reach extremes far from the optimal growth range, the culture may even collapse.

The influence of temperature on growth is partly explained by an alteration in the cell membranes. Cellular membranes change their fluidity in response to environmental temperature fluctuations, intending to maintain cell plasticity and enzymatic function [143]. High temperatures lead to an increased fluidity of the cellular membrane, which can ultimately disrupt the lipid bilayer [170]. Increased temperatures induce a decrease in photosynthetic activity and plastid membranes, more specifically, MGDG [8]. On the other hand, lower temperatures decrease the fluidity of the membranes, which also hampers electron transfer and the consequent function of important biochemical pathways like photosynthesis, nitrogen assimilation, and carbon fixation. Also here, lipid response is characterised by a decrease in MGDG content, and therefore a loss of plastid surface. Nevertheless, the decrease in MGDG content has been correlated to an increase in DGTS in *Nannochloropsis*, considered an indispensable trait for adaptation at low temperatures [8].

Lower temperatures are also related to an increase in PUFA content since the unsaturation of fatty acids increases the fluidity of the membrane [171,172]. In a different study, no net increase in the content of EPA was observed, suggesting differences between the short-term and long-term response of a culture at low temperature [8]. Regarding lipid classes, both MGDG and DGDG showed a net increase in PUFAs in their fatty acyl composition, compared to *Nannochloropsis* cells grown at optimal temperature. Also, DGTS increased the content of mainly EPA, showcasing a relevant role for this lipid in EPA trafficking at low temperatures [8,66]. How this lipid species contributes to maintaining homeostasis at low temperatures is still unclear. It was hypothesised that the large size of the polar head could contribute to maintaining a lipid bilayer structure, similar to DGDG in plastid membranes [66]. Other hypothesis highlights a storage role of PUFAs or a role in desaturation of PUFA intermediates [66]. Further studies with other microalgae species will help clarify the role.

Furthermore, temperature changes induce the enzymatic activation of carbon storage mechanisms [173]. At both sub- and supraoptimal temperatures, TAG content increased significantly at the expense of reduced growth rates [8]. Nevertheless, TAG content decreased at critical suboptimal growth temperatures due to a drastic reduction of photosynthetic activity, which limited both carbon accumulation and growth.

#### 6.1.2. Light

Light intensity above saturation limits (200–500 µmol photons m^−2^ s^−1^ depending on the light source and growth stage [174]) was shown to increase the neutral lipid content, particularly triacylglycerols (TAG), at the expense of polar lipids in *Nannochloropsis* [175,176]. This is because higher light intensities than saturation require fewer thylakoid membranes for light capture, and a reduction of plastid membrane surface occurs. The synthesis of TAG does not only come from recycling membrane lipids such as MGDG, but also from the fatty acid synthesis in the cytosol [175]. Nevertheless, exposure to extreme light conditions damages the photosynthetic antennas and their reaction centres, leading to the photobleaching of photosynthetic pigments and a decreased synthesis of TAGs [175,176]. The response of the phospholipids and betaine lipids at increased light intensities is less unanimous. Different studies showed a decrease in PC at high light intensities [175], while a different study did not observe any changes in phospholipid content [176]. In one study, the decrease in PC was concomitant to an increase in DGTS, hypothesising an interchangeable function between the two lipid classes [175]. However, no changes in DGTS content were observed in the other studies, emphasising the complexity of lipid remodelling in *Nannochloropsis*. At highly saturating light intensities, the PUFA percentage in PE, MGDG, and DGDG was shown to increase, most likely as a photoprotection mechanism to stand the oxidative conditions in the chloroplast [175,176]. Despite the percentage increase in EPA in those lipid species, the overall content also decreased due to damage of the plastid membranes [176].

On the other hand, light intensities below saturation increase the content of polar lipids and unsaturated fatty acids, including oleic acid, ARA, or EPA. Cells coping with low light intensity require a membrane architecture that maximises light capture (photoacclimation); therefore, a high surface area is accomplished by an accumulation of galactolipids, namely, MGDG [168,177,178].

Other process parameters, such as the light source (whole wavelength spectrum or a defined wavelength) and the light regime (continuous, pulse, light-dark cycles), were investigated in *Nannochloropsis* with a high variability of observations in PUFA accumulation. UV radiation (405 nm) and a mixture of red and blue light increased the PUFA content in *Nannochloropsis*, but red light alone decreased it [163,164]. Exposure to flashing light pulses (5 and 50 Hz) increased the PUFA content by 1.4- and 1.9-fold compared to a continuously illuminated culture [165]. The application of these technologies requires a precise match of the physiological characteristics of the culture, the light quality, or the regime. Investigating the lipidome together with the pigmentation could help in advancing the application of these technologies for lipid production in *Nannochloropsis*.

#### 6.1.3. Nutrient Availability

##### Nitrogen

Under nitrogen-replete conditions, the rate of photosynthetic carbon assimilation is seven to ten times higher than the rate of nitrogen assimilation in microalgae. During this phase, cells synthesise essential cellular components that contain nitrogen, including pigments and proteins, indirectly favouring the presence of membrane lipids.

Contrarily, nitrogen limitation comes with a decline of cellular photosynthetic capacity due to the degradation of Rubisco (ribulose-1,5-biphosphate carboxylase oxygenase). In terms of lipids, polar lipid contents are reduced, and their fatty acids are used for TAG synthesis. In the initial phases of nitrogen starvation, i.e., before the photosynthetic capacity is reduced, the fixation of carbon may surpass the demand for nitrogen assimilation. As a result, the surplus of carbon is redirected to the synthesis of storage compounds, such as storage carbohydrates and lipids [179,180]. Because of this interconnection between nitrogen assimilation and carbon metabolism, nitrogen limitation is a common technique used to boost neutral lipid production inside the cells. Green microalgae can have their lipid content increased by 2–3 times when they are cultured under nitrogen limitation for 4–9 days [181].

Compared to adverse light and temperature conditions, the levels of DGDG and DGTS are both reduced in the long term. In the short term, PG and MGDG are the two most sensitive lipids classes and the first ones to decrease in content [49]. From the different polar lipids, PE did not decrease under nitrogen starvation, implying a specific role for this lipid under nitrogen starvation [49]. The knockdown of the phospholipid DAG acyltransferase (PDAT) in N. Oceanica, responsible for trasnferring acyl moi-eties from glycerolipids to DAG, increased the contents of PE containing SFA. These results imply that PE could play a role as a transient carbon and electron sink and therefore contribute to protection from oxidative damage.

The fatty acid turnover between membrane and neutral lipids also has direct implications in the PUFA content. Also in this case, the decrease in glycolipids results in a decrease in PUFAs, more specifically EPA. A fraction of this polar EPA is mobilised to TAG, whose overall PUFA content in this fraction increased despite the composition being stable under nitrogen deprivation [182]. Nevertheless, a net loss of EPA occurs, indicating that EPA is not solely recycled but also used for other biological functions such as TAG synthesis.

##### Phosphorus

Phosphorus (P) is present in various biological molecules such as DNA, RNA, polyphosphates, phosphoproteins, sugar molecules, and cell membranes as phospholipids. Within the cell, phosphate is used in the form of adenosine phosphates or NADPH. Therefore, the insufficiency of P will impact various cellular energy-dependent processes, such as the carbon cycle, protein synthesis, and RNA transcription [65].

Under phosphorus limitations, microalgae undergo a process of lipid remodelling involving the degradation of P-containing lipids to accumulate P-free lipids [65,183]. During P limitation, the microalga *Nannochloropsis oceanica* continues producing PUFAs such as EPA, while there is a reduction in phospholipids such as PC. DGTS plays an active role in replacing PC in the desaturation of C18 fatty acids under phosphorus starvation conditions. Matsui studied the deficiency of phosphorus and changes in the composition and intracellular distribution of the fatty acids. In these conditions, the cells accumulated predominantly neutral lipids. The polar lipids of the cells showed higher contents of ARA and EPA during the initial period of the phosphorus deficiency. The study also showed that phosphorus deficiency triggers the increase in fatty acid desaturation in non-plastidial membranes [184].

#### 6.1.4. Two-Step Cultivation

In two-step cultivation, the bioactive compound of interest is triggered by the abrupt change in the cultivation environment or media. Several studies involving two-step cultivation were conducted to increase the levels of PUFAs. In one study, the first stage focused on optimising biomass growth, providing optimal cell growth conditions. Following this, the culture was harvested and transitioned to the second stage, which aimed for lipid induction. In that stage, the culture medium was nitrogen deficient, and the aeration rate was reduced to 0.5 L min^−1^. The air was enriched with 2% CO_2_ to prevent carbon limitation. As a result, a decrease in the percentages of EPA and LA was observed. This is due to the halt in microalgae growth under conditions of nitrogen deprivation, where the production of reserve lipids is prioritised over the biosynthesis of polar lipids [185].

Another two-step cultivation study of *Nannochloropsis* sp. led to a 3.4-fold increase in the EPA content [186]. In the first stage, the microalgae were grown at 25 °C, and with a light intensity of 100 µmol photons m^−2^ s^−1^. In the second stage, the temperature was reduced to 10 °C and the light to 30 µmol photons m^−2^ s^−1^. The first stage of cultivation took 18 days, and the second stage was made between days 19 and 22. The second stage led to an increase in the EPA content at the expense of reducing the biomass yield, overall lipid content and production efficiency. Remarkably, this study showed a higher increase in EPA in the phospholipid fraction instead of the glycolipid fraction, emphasising the synergy of light and temperature effects [186].

### 6.2. Microalgae Strain Improvement

Microalgae strain improvement refers to the process of enhancing desirable traits in microalgae by using different techniques such as random mutagenesis (RM), adaptive laboratory evolution (ALE) or targeted mutagenesis [187,188,189]. This review will focus on RM with physicochemical agents and ALE, since the use of genetic engineering for RM and targeted mutagenesis has strict limitations on the European food and feed markets, as indicated in Regulation (EC) 1829/2003 of the European Union. Microalgae improved via RM and ALE do not fall under the restrictions imposed on genetically modified organisms (GMO) since these techniques accelerate in the laboratory changes that occur in nature, and no exogenous DNA molecules are used.

Strain improvement can be used for various applications, including increasing a strain’s thermotolerance, adapting a strain to grow in complex media (e.g., wastewater), and increasing the contents of carotenoids, lipids, phenolic compounds, and amino acids, among others, while maintaining growth productivities.

#### 6.2.1. Random Mutagenesis

Random mutagenesis is a technique used for generating mutants by using physicochemical agents or, sometimes, molecular biological techniques. It is a well-established technique, robust, cost-effective, and easy to perform, and it does require prior knowledge of the metabolism of the microalga. RM also allows the selection of the desired attributes by applying selective pressure [187]. Because of these traits, it is a widely used technique to improve microalgae strains. RM has also some disadvantages: the misuse of the mutagenic agent can put the health of the operator in danger; the selection of the strains is not easy since there is the need for appropriate selection tools; the mutants can have genetic and phenotypic instability; and obtaining mutants with the desired phenotype can be hard because of the randomness of the mutations. Very often, mutations can cause the death of the organism or can be detrimental when compared to the original genotype due to loss of function [190,191,192].

##### Mutagenic Agent

Mutagenic agents can be grouped into two major groups: physical and chemical. The physical mutagenic agents include particle and electromagnetic radiation. Particle radiation includes, for instance, β- and α-particles and fast neutron treatment. In the electromagnetic radiation group, we can find mutagenic agents such as X-rays, UV light, and γ-rays [193].

Fast neutron treatment uses neutron bombardment or fast neutron irradiation to generate a library of mutants. Usually, this approach will create deletions in the genome that can go from 1 bp to 18 Mb. It also generates single-base substitutions. This method can generate stable mutants, and it has already been performed in microalgae with success. Fast neutron treatment can produce reactive oxygen species, which, in turn, can also cause mutations [194,195]. UV light acts on the DNA and can cause the formation of pyrimidine dimers, where a covalent bond is formed between a pair of thymines or cytosines [196]. γ-rays are also a form of nuclear mutagenesis that acts on the DNA molecules. The rays usually induce a glycosidic bond breakage or a base oxidation [197].

Chemical agents consist of chemicals that have various effects on the DNA of microalgae. Active oxygen species can cause oxidative damage in the DNA, namely, transversions, where, for example, a pyrimidine (thymine or cytosine) is substituted by a purine (adenine or guanine). Chemical mutagens can be grouped into three groups: (1) agents that chemically modify a base on the DNA; (2) agents that induce the formation of frameshifts, and (3) base analogues. In most cases, the culture must be growing actively for the mutation to become fixed in the genome. Ethyl methane sulphonate (EMS), one of the most used chemical mutagens, belongs to the first group. EMS is an alkylating agent and adds ethyl groups to different positions on all four bases, modifying their structure and leading to mispairing of the DNA during replication. For instance, the addition of an ethyl group to the oxygen-6 of guanine leads to a mispairing with thymine when it should have been with cytosine, resulting in a point mutation of the DNA from GC to AT [198]. Another example of an alkylating agent used in RM is N-methyl-N’-nitro-N-nitrosoguanidine (NTG), which adds methyl groups to the deoxynucleotides.

##### Selection with Pathway Inhibitors

One strategy to select mutants is by using pathway inhibitors. Different inhibitors have been used for the screening of lipid-enriched *Nannochloropsis* sp. mutants. *De novo* fatty acid synthesis is catalysed by four main enzymes: acetyl-CoA carboxylase (ACC), malonyl-CoA-acyl carrier protein (ACP) transacylase (MAT), fatty acid synthase (FAS), and fatty acyl-ACP thioesterases [33]. Therefore, changes in the expression of these enzymes have an impact on fatty acid synthesis. Because of this, growth inhibitors targeting the expression of these enzymes have been chosen for the selection. The principle is to limit the wild-type growth so that only mutants with higher expression of the enzymes survive. In that way, higher levels of FA will be found in the mutants under non-selective conditions. The formation of malonyl-CoA by ACC usually represents one of the rate-limiting steps for de novo synthesis of FA [199]. Studies with different selection growth inhibitors tested on *Nannochloropsis* sp. can be found in Table 5.

**Table 5:** Growth inhibitors used in previous studies to isolate lipids-enriched mutants of the microalgae *Nannochloropsis* sp.

Growth Inhibitor	Concentration	Improvement	Study
Cerulenin	25 μM	29% EPA increase	Chaturvedi et al. [200]
Cerulenin and Galvestine-1	50/60 μM and 10 μM	Increased membrane lipids and EPA 1.4-fold	Razali et al. [201]
DCMU ^1^	2 μM	EPA increase	Zhang et al. [202]
Erythromycin	50 μg/mL	12% EPA increase	Chaturvi et al. [200]
Quizalofop	50 and 70 μM	PUFA, EPA, and TFA increase	Chaturvi et al. [203]

^1^ 3-(3, 4-dichlorophenyl)-1,1-dimethylurea.

Cerulenin is an antibiotic that acts on the FAS complex, and the inhibitory effect is imposed on the ketoacyl carrier protein synthase (KAS) isoform [200]. DCMU blocks the electron transfer at the reducing site of photosystem II, also altering other cell processes such as chlorophyll and fatty acid synthesis and cyclic phosphorylation [202,204]. In turn, galvestine-1 is a chemical capable of inhibiting the MGDG [201,205], whereas erythromycin is an antibiotic that targets protein synthesis and causes damage in the chloroplast through the inhibition of the photosynthetic electron transport, which has implications in the photosystems I and II of microalgae [206,207]. Finally, quizalofop is an herbicide that targets the ACC. Mutants resistant to quizalofop might have a different binding site configuration, making them resistant to this herbicide [203].

Some of these growth inhibitors are light-sensitive, and since *Nannochloropsis* sp. are photoautotrophic organisms, the use of these photosensitivity-inducing inhibitors can give rise to false positives. Another drawback of this selection process is that there is still a gap in the knowledge of the action mechanism of lipid inhibitors in microalgae. This is because most of the knowledge of these inhibitors comes largely from plant studies, and some crucial differences might exist.

#### 6.2.2. Adaptive Laboratory Evolution

Adaptive laboratory evolution (ALE) is a way to improve a desired trait of a microorganism by applying selective pressure for a prolonged time [208]. When a population is under long-term selective pressure, the individuals that are more adapted to those conditions, i.e., the fittest, will thrive and pass those genes more often to the new generations [209]. Evolution is currently seen as a two-step process that takes place continuously over time: the first step involves the random generation of a variation because of mutations and recombination; and the second step involves the non-random survival and reproduction of the fittest individuals for the environment. These two steps occur continuously over time, leading to the evolution of the populations towards the fittest under a specific set of conditions [190].

Usually, in ALE experiments, there are mutations in stress response genes. Under stressful conditions, the stress response genes are activated at the expense of housekeeping genes and growth functions. If the stressful conditions are withdrawn, the stress response genes are deactivated, and the cell resumes its normal activity. Experimental evolution keeps the stressful conditions constant from one generation to the next, allowing for the stress response to remain active. Under this constant selective pressure, any mutation that frees up energy from the stress response to enhance growth is likely to be favoured. This also suggests that selection in an environment that alternates between stress and no stress would lead to fewer mutations than in a constant stressful environment [210].

ALE experiments on microalgae present some advantages: large populations, short generation time, easy environmental control, and the possibility of having populations cryopreserved and later revived. The latter advantage means that the researcher can compare different populations within the same experiment and offers the possibility of a restart when an experiment fails. ALE on microalgae also has some disadvantages: in most cases, microalgae perform asexual reproduction, and since there is no recombination of alleles, the population will present a lower genetic variation, being dependent mostly on spontaneous beneficial mutations to evolve. Another downside of this type of experiment is that some evolutionary processes can be very long [211]. Up to now, the authors only know about one ALE study made with *Nannochloropsis*, cited in Table 6.

**Table 6:** Strain improvement studies that were made on microalgae, with a focus on *Nannochloropsis* sp. and lipid content. The abbreviations used in the following table are ALE, adaptive laboratory evolution; EMS, ethyl methane sulphonate; NTG, nitrosoguanidine; RM, random mutagenesis; and UV ultraviolet light.

Microalgae	Strain Improvement Method	Mutagenic Agent/Selective Pressure	Improvement	Study
*Nannochloropsis gaditana*	RM	EMS	Increased productivity	Perin et al. [212]
*Nannochloropsis gaditana*	RM	EMS	Increased photosynthetic activity and productivity; decreased chl content	Perin et al. [213]
*Nannochloropsis gaditana*	RM	EMS	Increased lipid productivity	Cecchin et al. [214]
*Nannochloropsis gaditana*	RM	EMS	Increased lipids and ketocarotenoid productivity	Cecchin et al. [215]
*Nannochloropsis oceanica*	RM	Heavy ion irradiation	Increased growth rate, chl-*a* content, and lipid productivity	Ma et al. [70]
*Nannochloropsis oceanica*	RM	EMS and NTG	Increased lipid productivity	Wang et al. [216]
*Nannochloropsis oceanica*	RM	Nuclear radiation	Increased biomass productivity and higher oxygen evolution rate	Lu et al. [217]
*Nannochloropsis oculata*	RM	DCMU	Increased EPA	Jimin et al. [204]
*Nannochloropsis oculata*	RM	MNU	Increased PUFA, EPA, and TFA	Chaturvedi et al. [205]
*Nannochloropsis oculata*	RM	UV 320–400 nm	Higher lipids: chl	Srinivas et al. [218]
*Nannochloropsis oculata*	RM	UV 345 nm	Higher lipid content; increased ω-3 and ω-6	Moha-Léon et al. [219]
*Nannochloropsis oculata*	RM and ALE	EMS and temperature stress	Increased temperature tolerance by 10 °C and lipid productivity and content	Arora et al. [220]
*Nannochloropsis oculata*	RM	EMS	Increased PUFA, carbohydrate, and pigment productivity	Arora et al. [221]
*Nannochloropsis oculata*	RM	EMS	Increased membrane lipids and EPA content	Razali et al. [201]
*Nannochloropsis oculate ST-6*	RM	EMS	EPA increase	Chaturvedi et al. [200]
*Nannochloropsis* sp.	RM	EMS	Increased lipid productivity	Anandarajah et al. [222]
*Nannochloropsis* sp.	RM	EMS	TFA increase; PUFA decrease	Doan et al. [162]

## 7. Future Perspectives

*Nannochloropsis* species is a model and industrial microorganism for the marine biosynthesis of lipids and a unique photoautotrophic source of PUFA, especially EPA. In the last decades, the main study focus of *Nannochloropsis* species was on the accumulation and optimisation of triacylglycerols for fuel applications. However, the high EPA content of *Nannochloropsis* sp. (up to 5% *w*/*w*) has positioned its biomass as a valuable source for feed applications in niche markets, while also opening the possibility to use it for food, cosmetic, and pharmaceutical applications.

Until now, the bioactivity of EPA has been mainly explored independently of the hosting lipid class, and further research on the entire molecule might open a horizon of new opportunities in the market. The advancement of lipidomics in combination with other -omics techniques will contribute to that purpose by making the analysis of lipid classes more accessible, faster, and more cost-effective. A better understanding of the lipidome of *Nannochloropsis* species and the biosynthetic pathways of EPA under both optimal and adverse growth conditions is still necessary. This information is indispensable for cultivation control at a large scale, the definition of targets for genetic engineering, or the finding of a selective pressure for the isolation of the phenotype of interest. Moreover, further elucidation of the lipidome will fill the knowledge gaps concerning the biological role and involvement of different glycerolipids involved in EPA biosynthesis, such as DGTS.

In the case of non-targeted approaches, screening procedures are still required to mark EPA and subsequently sort the cells with the desired phenotype. Currently, there are no methods that can provide a fast, non-invasive, simple, and accurate assessment of *Nannochloropsis* EPA content in real-time. A possible advancement would be the combination of vibrational spectroscopy methods, such as Raman or FTIR, as well as fluorescence spectroscopy linked to flow cytometry and cell sorting. As well as screening phenotypes of interest, these techniques are useful for lipid monitoring during cultivation and therefore help in process optimization (f.e. defining optimal harvest time). Finally, most of the classic mutagenesis approaches with *Nannochloropsis* sp. referenced in the literature were only validated at the laboratory scale. Future studies ought to validate their performance at an industrially relevant scale after confirming the stability of the phenotype. Overall, the development of clear guidelines for the mutagenesis, screening, validation, and scale-up could facilitate the development of phenotypes of *Nannochloropsis* and other microalgae of industrial interest. Also, adaptive laboratory evolution, in studies with different microalgae species, proved to be a strong tool in adapting a strain to a specific environment to favour a set of phenotypic traits. Possible selective pressures favouring cells with increased PUFAs include low temperatures or specific lipid inhibitors such as herbicides, despite more fundamental knowledge on the action mechanism of herbicides still being needed. ALE experiments on *Nannochloropsis* sp. under such stress factors could help in the isolation of populations with higher PUFA contents.

## Figures and Tables

**Table 1:** Contents of ω-3 and ω-6 fatty acids in plant and animal-based foods. The values were obtained from https://fdc.nal.usda.gov/ on 17 November 2024 except when indicated otherwise.

	Food	ω-3	ω-6	
		EPA	LA	ARA
Dairy	Butter ^1^	0.024	2.25	0.104
	Cheddar	0.01	0.939	0.051
	Eggs	*	1.46	0.02
	Greek Yogurt ^2^	-	0.01	-
	Parmesan	0.008	0.999	0.027
	Whole milk	0.001	0.097	0.004
Fish	Bluefin tuna ^3^	0.252	0.06	-
	Haddock ^4^	0.042	0.014	0.009
	Pollock ^4^	0.049	0.005	0.005
	Salmon ^3,4^ [92]	0.4	0.1	-
	Tuna ^5^	0.025	0.013	0.022
Fish oils	Cod liver	6.9	0.935	0.935
	Salmon	13	1.54	0.675
	Sardine	10.1	2.01	1.76
Legume products	Hummus	-	6.81	0.005
	Peanut butter	*	9.73	*
	Soy milk	-	0.988	-
Meat	Beef loin ^6^	0.002	0.362	0.064
	Chicken breast ^7^	0.004	0.599	0.086
	Cured bacon ^6^	0.003	5.27	0.16
	Ham	-	0.446	0.053
Oils	Coconut	-	1.68	-
	Olive	0.001	9.74	0.044
	Sunflower	-	13	0.02
Vegetables	Broccoli ^8^	*	0.017	-
*Nannochloropsis*	Whole biomass (dry)	2.24	0.36	0.69
*Nannochloropsis* extract	Fish feed ingredient (NannoStarGOLD, AlgaSpring)	13	*	1.8
*Nannochloropsis*-enriched oil	Food supplement (OMEGA-3, Iwi)	25	*	*
	Food supplement (Almega PL^®^, Qualitas Health Lda)	2.3	50.8	*

Values in g/100 g; * no information; ^1^ salted; ^2^ nonfat; ^3^ wild; ^4^ raw; ^5^ canned, in water; ^6^ cooked, roasted; ^7^ cooked, braised; ^8^ raw.

**Table 3:** Summary of the online and offline methods for lipid detection and analysis.

Methods	Processing Time	Principle	Lipid Class Target	Accuracy	Scale	Application
Spectrofluorometry	Off- and online	Fluorescence	NL; PL; EPA	Medium/High	Laboratory	LQM
Flow cytometry	Off- and online	Fluorescence	NL; PL	High	Laboratory	HTS and LQM
Fatty Acid Methyl Ester (FAME) analysis	Offline	Chromatography	NL; PL; TFA	High	Lab- to commercial	SA
Iodine value (IV)	Offline	Titration/Spectroscopy/Chromatography	NL; PL; TFA	Medium/High	Lab- to commercial	SA
Mass spectrometry (GC-MS; DI-MS; LC-MS)	Offline	Electron ionisation	NL; PL; PUFA	Medium/High	Laboratory	SA
NIR-FTIR	Off- and online	Spectroscopy	PUFA	High	Laboratory	LQM
Raman spectroscopy	Off- and online	Spectroscopy	PUFA	High	Laboratory	HTS and SA
Solvatochromism	Off- and online	Absorbance/Fluorescence	NL, PL	Medium/High	Laboratory	HTS
Tetrazolium (TTC) assay	Offline	Colorimetry	PUFA	Low	Laboratory	HTS
Sulpho-phospho-vanillin (SPV) assay	Offline	Colorimetry	NL; PUFA	Low/Medium	Laboratory	HTS
TerHz	Offline	Spectroscopy	TFA	High	Laboratory	SA

The abbreviations used in the table are as follows: DI-MS, direct infusion–mass spectrometry; EPA, eicosapentaenoic acid; HTS, high-throughput screening; GC-MS, gas chromatography–mass spectrometry; LC-MS, liquid chromatography–mass spectrometry; LQM, lipid quality monitoring; NL, neutral lipids; PL, polar lipids; PUFA, polyunsaturated fatty acid; SA, sample analysis; TFA, total fatty acids.

**Table 4:** *Nannochloropsis* sp. lipid classes composition measured by two different methods. The abbreviations used in the table are the following: LC-MS/MS—liquid chromatography–mass spectrometry/mass spectrometry; TLC-GC/FID—thin layer chromatography–gas chromatography/mass spectrometry. The values from LC-MS/MS are an approximation since they were read on a graph.

Lipid Class	Condition	Low Light		High Light	
	Method	LC-MS/MS [49]	TLC-GC/FID [8]	LC-MS/MS [49]	TLC-GC/FID [8]
Diacylglycerol–trimethyl–homoserine (DGTS)		0.8	0.7	0.4	0.6
Digalactosyldiacylglycerol (DGDG)		1	1.4	0.9	0.9
Monogalactosyldiacyglycerol (MGDG)		3.2	2.6	1.5	0.5
Phosphatidylcholine (PC)		1.9	1.4	0.6	1.2
Phosphatidylethanolamine (PE)		0.4	0.2	0.4	0.3
Phosphatidylglycerol (PG)		3.2	0.8	1.5	0.5
Phosphatidylinositol (PI)		0.5	0.4	0.2	0.3
Sulphoquinovosyldiacylglycerol (SQDG)		1.8	1	2.4	0.6
Triacylglycerols (TAG)		0.2	2.3	11.4	10.1

## Data Availability

No additional data was generated.
